# A Few Things to Be Remembered

**Published:** 1894-04

**Authors:** L. P. Haskell


Article vii.

### A FEW THINGS TO BE REMEMBERED.

BY L. P. HASKELL.

In ninety-nine per cent. of mouths the centre of the palate is hard and unyielding, in fact the only portion of the upper jaw which does not change from absorption or yield to pressure. Unless provision is made for it, the plate will, sooner or later, rock. This should be remedied by a "relief" in metal plates, of a thin film of wax on the model, extending well up on the anterior portion to near the margin of the process, and to within a quarter of an inch of the rear of plate. In a rubber plate the relief can be made by burring or scraping the plate.

There are more failures in artificial dentures from *faulty articulation* than from any other cause. To guard against this, in adjusting a denture in the mouth, see to it that none of the six anterior teeth touch,—in fact leave a margin of space. This will prevent the tilting of the plate from the rear. Be sure the bicuspids and first molars on both sides meet uniformly; have no pressure on the second molar, and especially if the lower occluding molar leans forward, as it would crowd the denture forward.

In arranging the *lower* teeth, commence with the second bicuspid so as to ensure a perfect interlocking of the cusps. The fronts must be accommodated to the space allotted to them by crowding or overlapping, if needed.

In ordering teeth from the dealer, see that bicuspids and molars are provided that have a good length of porcelain *above* the pins, so that if necessary to grind, in articulating, the porcelain will not be ground away. The teeth will also present a more natural appearance. Insist upon this from your dealer.

If you desire to restore the expression of the mouth which has been sacrified by the extraction of the cuspid teeth, remember this invariable rule, viz.: the plate can and should be worn higher over these teeth than elsewhere, and the artificial gum made fuller.

Leave the necks of the cuspids slightly fuller than the other teeth.

Finish the rubber with a festoon around the necks of the teeth,

In selecting teeth for metal plate and crown work, if you desire *strength,* use the perpendicular rather than the cross-pins, and they are less liable to crack in soldering, and do not let your dealer give you anything else.

In polishing metal work, use *oil* with your pumice both on the felt and the brush. To reach all the depressions and interstices, drive a pine stick into the lathe chuck made for it and with sharp knife turn it to a blunt point.
*Ohio Dental Journal.*

